# Case report: Neuronal intranuclear inclusion disease presenting with acute encephalopathy

**DOI:** 10.3389/fneur.2023.1184612

**Published:** 2023-06-02

**Authors:** Julia Ting Bu, Dolores Torres, Adam Robinson, Corey Malone, Juan Carlos Vera, Shadi Daghighi, Anastasie Dunn-Pirio, Suzan Khoromi, Justin Nowell, Gabriel C. Léger, Joseph D. Ciacci, Vanessa S. Goodwill, Melanie Estrella, David G. Coughlin, Yueyang Guo, Nikdokht Farid

**Affiliations:** ^1^Department of Neurosciences, University of California, San Diego, La Jolla, CA, United States; ^2^Department of Radiology, University of California, San Diego, La Jolla, CA, United States; ^3^Sharp Rees-Stealy, Department of Radiology, San Diego, CA, United States; ^4^Department of Radiology, State University of New York Upstate Medical University, Syracuse, NY, United States; ^5^Sharp Rees-Stealy, Department of Neurology, San Diego, CA, United States; ^6^Department of Pathology, University of California, San Diego, La Jolla, CA, United States

**Keywords:** neuronal intranuclear inclusion disease, magnetic resonance imaging, arterial spin labeling, chromatolysis, case report

## Abstract

Neuronal intranuclear inclusion disease (NIID), a neurodegenerative disease previously thought to be rare, is increasingly recognized despite heterogeneous clinical presentations. NIID is pathologically characterized by ubiquitin and p-62 positive intranuclear eosinophilic inclusions that affect multiple organ systems, including the brain, skin, and other tissues. Although the diagnosis of NIID is challenging due to phenotypic heterogeneity, a greater understanding of the clinical and imaging presentations can improve accurate and early diagnosis. Here, we present three cases of pathologically proven adult-onset NIID, all presenting with episodes of acute encephalopathy with protracted workups and lengthy time between symptom onset and diagnosis. Case 1 highlights challenges in the diagnosis of NIID when MRI does not reveal classic abnormalities and provides a striking example of hyperperfusion in the setting of acute encephalopathy, as well as unique pathology with neuronal central chromatolysis, which has not been previously described. Case 2 highlights the progression of MRI findings associated with multiple NIID-related encephalopathic episodes over an extended time period, as well as the utility of skin biopsy for antemortem diagnosis.

## Introduction

Neuronal intranuclear inclusion disease (NIID) is a genetic progressive leukoencephalopathy with multiple clinical presentations that make prompt diagnosis challenging in many cases (1). Clinical presentations have been classified in a variety of methods including age of onset (infantile-onset, juvenile-onset, and adult-onset subgroups), family history (sporadic or familial patterns), and affected areas of the nervous system (the central nervous system, peripheral nervous system, or autonomic nervous system predominant subtypes) (1). Adult-onset cases can be sub-grouped into dementia-dominant and limb weakness-dominant phenotypes (2). Infantile-onset cases are marked by cerebellar findings of ataxia and dysarthria occurring before 5 years of age. Juvenile-onset cases are marked initially by behavior changes and then later the onset of pyramidal and cerebellar signs (3). Sporadic cases of NIID most commonly exhibit dementia, ataxia, autonomic dysfunction, and parkinsonism, whereas familial cases are more likely to be associated with muscle weakness, sensory disturbances, and juvenile onset (2). NIID is the second most common adult-onset genetic leukoencephalopathy, second to CADASIL (4). NIID is pathologically characterized by extensive intranuclear eosinophilic inclusions that are ubiquitin and p-62 positive on immunohistochemical staining affecting multiple organ systems including the brain and skin (1, 3, 5). Recently, a CGG repeat expansion in a non-coding region of *NOTCH2NLC* on chromosome 1 has been discovered (6) as a causative gene mutation leading to NIID.

One of the characteristic imaging features of NIID is the magnetic resonance (MR) imaging finding of hyperintense signal in the corticomedullary junction on diffusion-weighted imaging (DWI). A recent case report also noted high DWI in globus pallidus, suggesting that deep gray matter nuclei can also be affected (7). Other common MRI features include white matter changes, vermian T2 hyperintensities, and focal brain edema. Thus, the radiographic appearance of NIID has significant overlap with other leukoencephalopathies but also vascular, infectious, and toxic etiologies as well. Given the growing awareness of NIID and less invasive methods of identifying the pathognomonic inclusions through a skin biopsy, we reviewed three recent pathologically proven adult-onset dementia-predominant cases of NIID to illustrate the clinical and imaging heterogeneity of these cases along with novel imaging and pathological features. All cases presented with episodes of acute encephalopathy with periodic protracted hospitalizations and extensive workups, and all spanned several years between symptom onset and definitive diagnoses. Case 1 provides a striking example of ASL hyperperfusion in the setting of acute encephalopathy and also exhibits unique pathology with prominent neuronal central chromatolysis. Case 2 highlights the progression of MRI findings associated with multiple NIID-related encephalopathic episodes over an extended time period, as well as the utility of skin biopsy for antemortem diagnosis.

## Case descriptions

### Case 1

**Figure 1:**
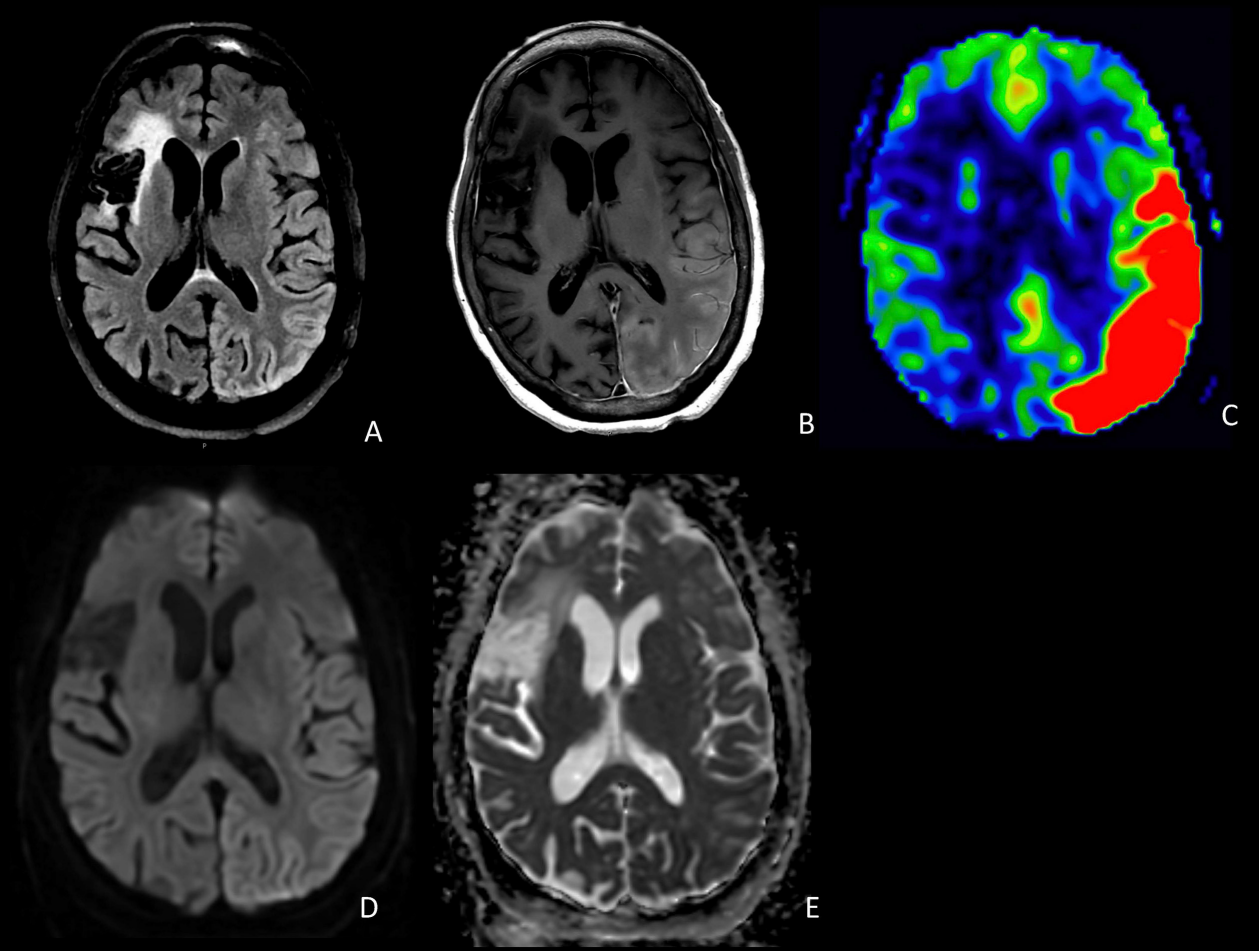
Axial FLAIR **(A)**, T1 post-contrast **(B)**, and ASL **(C)** demonstrate cortical edema, cortical and pial enhancement, and marked hyperperfusion involving the left temporal and occipital lobes. DWI **(D)** and ADC **(E)** show T2 shine-through but no definite restricted diffusion in the involved regions. Also noted is a chronic infarct in the right frontal lobe.

**Figure 2:**
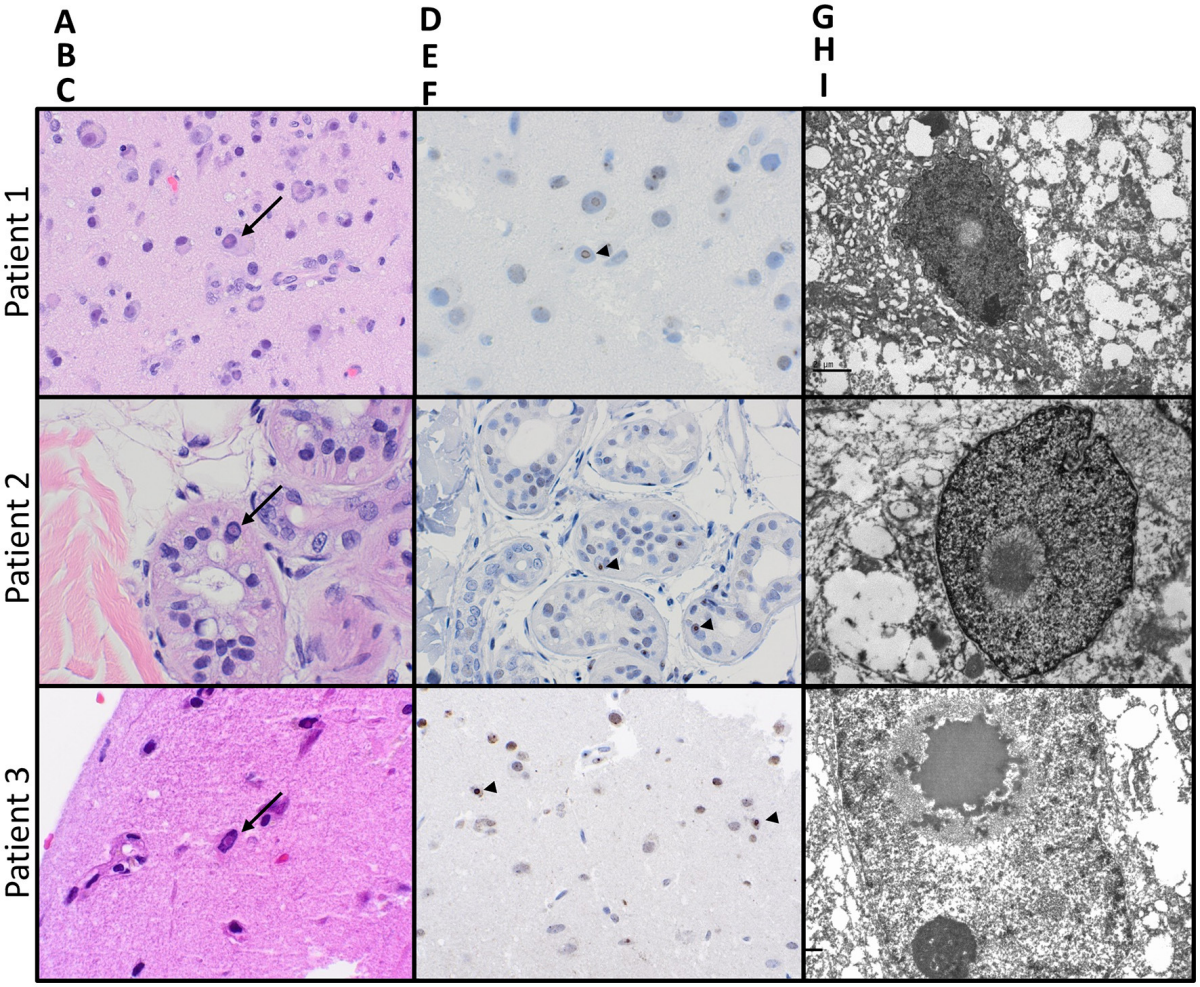
Pathologic features of NIID on biopsies from all three patients: Hematoxylin- and eosin-stained sections [**(A–C)**; 400x magnification] from biopsies on all three patients showed prominent intranuclear eosinophilic inclusions (arrows), seen within neurons also exhibiting central chromatolysis in the brain biopsy from patient 1, the eccrine glandular epithelium on the skin biopsy from patient 2, and neurons and glia on brain biopsy from patient 3. All intranuclear inclusions were immunoreactive for ubiquitin [**(D–F)**; arrowheads]. Electron microscopy on all three specimens confirmed the presence of intranuclear fibrillar inclusions **(G–I)**.

A 59-year-old Hispanic woman with a history of right MCA stroke 3 years prior complicated by localization-related epilepsy, as well as a history of systemic sclerosis, was admitted to the hospital for acute-onset encephalopathy and global aphasia. Before 15 days, she received her first COVID-19 vaccine (Moderna) followed by the seasonal influenza vaccine. At presentation, she was febrile to 101.2°F. MRI of the brain did not show acute changes. Continuous EEG showed diffuse slowing and epileptiform discharges over the right posterior quadrant without seizures. Empiric antibiotics and antiviral medications were started for presumed meningitis or encephalitis. After 4 days, she was found to have a right facial droop involving the forehead and right upper extremity hemiparesis. A repeat MRI was again negative for acute infarct. Lumbar puncture showed mildly increased protein at 55 mg/dL, < 5 WBCs per mm^3^, matching oligoclonal bands between the CSF and serum, and normal cytopathology and flow cytometry, but elevated CSF IL-6 to 67.6 pg/mL and soluble IL-2R cytokines to 29.2 pg/ml. An extensive laboratory workup was largely unremarkable for toxic, metabolic, and infectious etiologies (see Supplementary material). On hospital day 8, she underwent a repeat MRI brain demonstrating new left hemispheric gyral swelling and edema involving the occipital, parietal, temporal, and posterior frontal lobes with cortical/pial enhancement and marked hyperperfusion throughout that region (Figure 1). Empiric steroids were not administered given the concern of possibly provoking a scleroderma renal crisis, and she was treated with a course of intravenous immunoglobulin 2 mg/kg and antiepileptic medications. There was no clinical improvement and a repeat MRI on hospital day 12 found worsened left hemispheric gyral swelling and enhancement but decreased hyperperfusion. Clinically, there was a concern for possible adult-onset mitochondrial encephalopathy, lactic acidosis, and strokelike episodes (MELAS), and she was treated with intravenous arginine, alpha lipoic acid, taurine, levocarnitine, vitamin C, and coenzyme Q10. Ultimately, she underwent a brain biopsy on hospital day 13, which showed neuronal eosinophilic intranuclear inclusions, immunoreactive for ubiquitin, consistent with NIID (Figure 2). Additionally noted on biopsy was prominent neuronal central chromatolysis, where neurons appeared enlarged with Nissl substance pushed to the cytoplasmic periphery. A repeat MRI on hospital 14 showed persistent gyral swelling and edema but the resolution of enhancement and normalized perfusion. Repeat lumbar puncture on hospital day 22 was notable for normalized cytokines and protein. On day 25 in the hospital, she was speaking in three- to five-word sentences. Upon follow-up, 3 months after discharge, there was a significant improvement in her aphasia and encephalopathy with only minor residual deficits. A follow-up MRI 6 months after the acute episode revealed resolved cortical swelling with residual encephalomalacia and gliosis.

### Case 2

**Figure 3:**
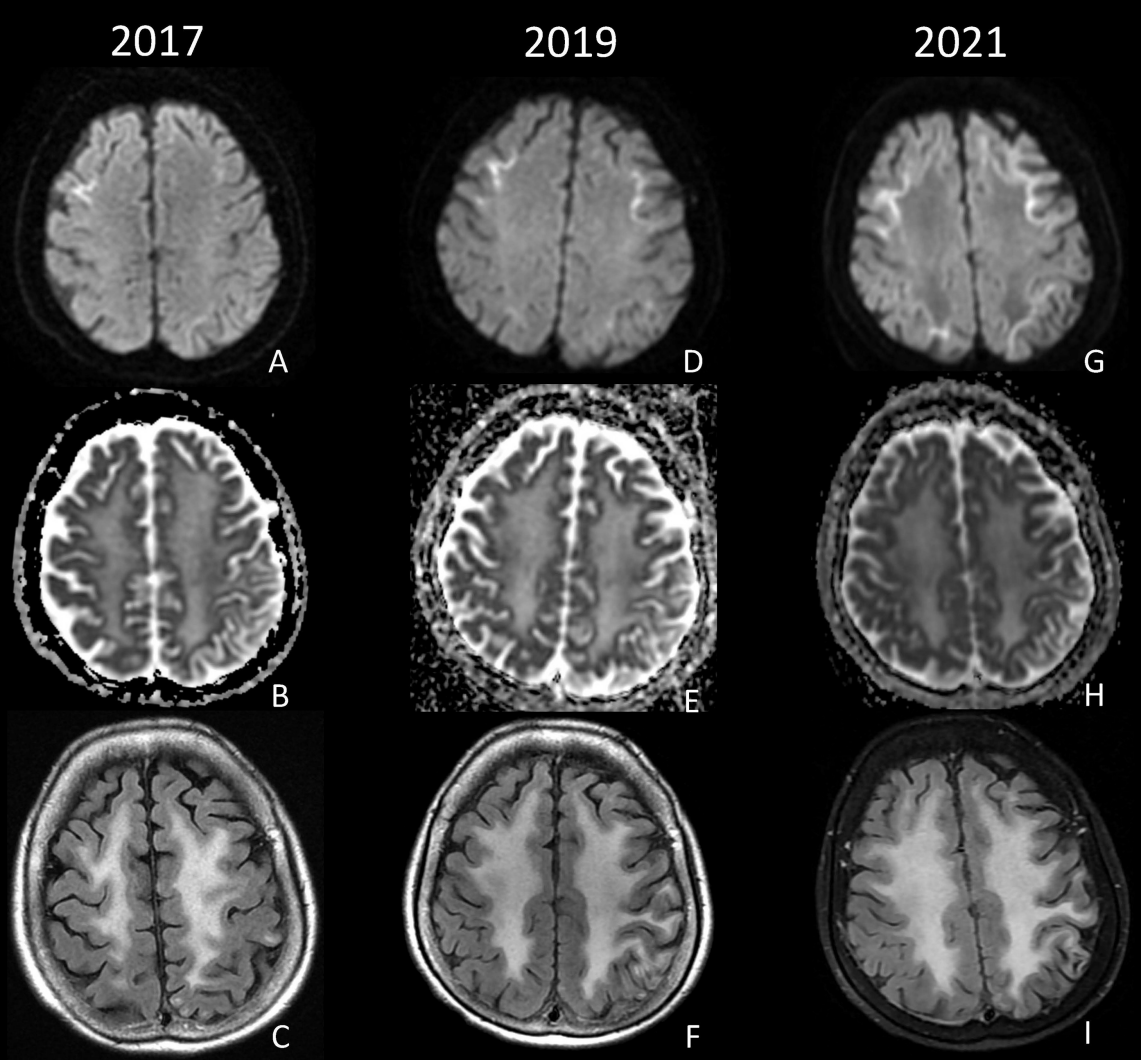
DWI, ADC, and FLAIR from 2017 **(A–C)**, 2019 **(D–F)**, and 2021 **(G–I)** demonstrate progressive diffusion restriction at the corticomedullary junction of the bilateral frontal and parietal lobes, as well as diffuse confluent white matter FLAIR hyperintensity.

A 60-year-old Asian female with hypertension presented after being found unconscious with unknown last known normal. She was noted to be encephalopathic with left-sided weakness and global aphasia. MRI brain showed elevated DWI restriction in the bilateral frontal lobes centered at the corticomedullary junction and confluent white matter T2 and FLAIR hyperintensity. LP CSF 14-3-3, PRP RT-QuIC, and West Nile Virus IgM were negative. Clinically, the patient gradually improved, and 2 years after the initial hospitalization, she was able to resume gardening and dancing, but the global aphasia persisted. She continued to have intermittent episodes of acute encephalopathy marked by confusion and aphasia requiring hospitalizations at years 4, 5, 6, and twice in year 7 after the initial presentation. Spinal fluid testing at years 4 and 6 post-initial hospitalization was notable for positive 14-3-3 protein with negative PRP RT-QuIC; T-tau 869 pg/mL; WBC < 5/mm^3^; and protein 40 mg/dL. Tests, such as CSF VDRL, coccidioides antibodies, cryptococcus antigen, HSV PCR, oligoclonal bands, flow cytometry, and paraneoplastic autoantibody panels, were all negative or normal. Repeat brain MRIs revealed progressively increasing symmetric DWI signal at the corticomedullary junction, extending posteriorly from the frontal lobes to involve the parietal and occipital lobes (Figure 3). Extensive genetic testing was unrevealing (see Supplementary material). Patient and family consistently declined brain biopsy; however, skin biopsy was obtained 7 years after the initial presentation which showed eosinophilic ubiquitin-positive intranuclear inclusions within the nuclei of eccrine sweat glands and adipocytes, consistent with NIID (Figure 2). The patient was discharged home with family support, and on a follow-up visit 2 months after admission, she was noted to be back to baseline with expressive aphasia, otherwise able to perform most of her activities of daily living and gardening.

### Case 3

**Figure 4:**
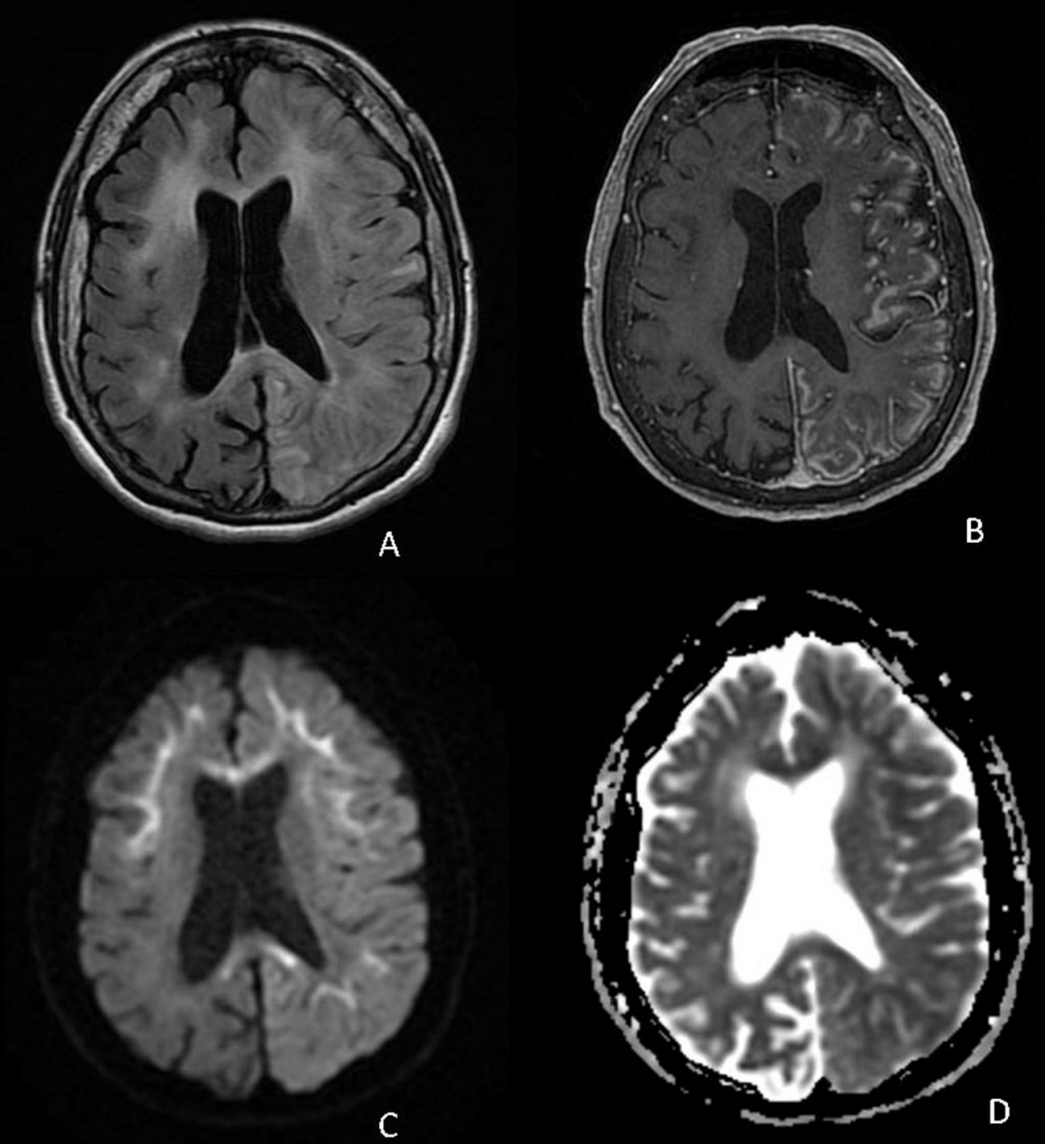
Axial FLAIR **(A)**, T1 post-contrast **(B)**, DWI **(C)**, and ADC **(D)** demonstrate diffuse cortical edema and patchy cortical enhancement throughout the left cerebral hemisphere on a background of corticomedullary diffusion restriction involving the bilateral frontal and left parietal lobes as well as diffusion restriction in the corpus callosum.

A 72-year-old Hispanic woman with ~8 years of progressive cognitive decline presented to the Emergency Department with confusion and lethargy. She was found to be febrile to 103.5°F, urinary retention, and with leukocytosis. In the preceding 5 years, she had multiple emergency room visits for episodes of confusion, dysarthria, headache, and fever thought to be secondary to urinary tract infections or TIAs and was noted by family to have had a progressive cognitive decline in between events. MR imaging demonstrated T2 hyperintensities with corresponding diffusion restriction raising concern for acute to subacute ischemia at several of these hospitalizations. At her fourth hospital admission, due to ongoing encephalopathy despite empiric antibiotics for the urinary tract infection, an expanded infectious workup was performed including a lumbar puncture. CSF studies were notable for neutrophilic pleocytosis (corrected WBC count 463 cells/mm^3^), positive CSF HSV IgG, negative CSF cultures, and negative CSF autoimmune panel. She underwent treatment for presumed HSV encephalitis without clinical improvement. On hospital day 9, a repeat brain MRI demonstrated diffuse left hemispheric cortical enhancement. On hospital day 20, a follow-up brain MRI demonstrated worsening, predominantly left hemispheric, cortical enhancement, as well as gyral swelling and edema (Figure 4). A brain biopsy was performed which confirmed the diagnosis of NIID. Electron microscopy revealed scattered intranuclear inclusions, mostly filamentous (Figure 2). She subsequently underwent high-dose pulse steroid therapy (hospital days 22–26) and plasmapheresis (four pheresis treatments from hospital days 28–34) with clinical improvement, alert to surroundings but still with limited response. A brain MRI performed on hospital day 26 demonstrated resolving left hemispheric gyral swelling and edema and cortical enhancement. Post-hospitalization she remained minimally conversive. She was transitioned to hospice care and passed away shortly at age 72, ~8 years after her initial symptom onset.

## Discussion

Neuronal intranuclear inclusion disease is marked by widely heterogeneous clinical presentations. However, among cases of adult-onset NIID, episodic attacks of acute encephalopathy appear to be an important diagnostic indicator. The cases in this series illustrate this presentation mimicking encephalitis in their CSF and imaging characteristics against a background of progressive dementia syndrome (1, 2). These patients had protracted periods of time that passed before definitive diagnoses could be made and underwent a number of procedures and treatments that potentially could have been avoided with more prompt recognition of their diagnosis. Two of the three cases had classical corticomedullary DWI restriction on brain MRI which is a helpful indicator, but the cases shown here also exhibit different changes that can occur during acute encephalopathic episodes including cortical swelling, contrast enhancement, and hyperperfusion. Spinal fluid sampling can show pleocytosis and inflammatory cytokine changes during and after encephalitic attacks. Case 1 is the first report of cytokine changes in NIID during an encephalitic attack. While it is not clear what provokes these attacks, the patient in case 1 had recently received COVID-19 and influenza vaccinations 2 weeks prior to presentation and immunological stress may be related to her presentation.

Case 2 also has a significant elevation in CSF 14-3-3 protein on testing, which likely reflects the acute neuronal injury caused by the encephalitic attack, as the more specific CSF PRP RT-QuiC test did not show indications of Creutzfeldt–Jakob disease. Elevations in 14-3-3 have been documented in other non-prion conditions such as acute neuronal injury (8) and Alzheimer's disease (9).

Case 3 also had a pleocytosis on presentation, and infectious encephalitis was initially on the differential. Previous publications have noted mild elevations in CSF protein and mild or no CSF pleocytosis in patients with NIID, even during encephalitic attacks (2, 5). Although no causative infectious agents were identified in case 3′s workup, we cannot fully exclude that she had a CNS infection that unmasked or worsened her underlying NIID and was the primary cause for her CSF pleocytosis, and it is less likely given that clinical improvement was noted after immunosuppressive treatment.

Previous cases of NIID demonstrating ASL hyperperfusion have been reported (10, 11). Interestingly, at least two cases of NIID with associated hypoperfusion have also been reported (12, 13). Pathologically, NIID is marked by eosinophilic inclusions in neurons, which are immunoreactive for ubiquitin or p62, and appear filamentous by electron microscopy. Interestingly, the biopsy from Case 1 also demonstrated prominent central chromatolysis, a histologic feature that may be seen in traumatic injuries or pellagra, but which has not been previously reported in NIID. It is now known that the pathognomonic intranuclear inclusions of NIID are present in peripheral tissues. Vascular smooth muscle cells have been shown to harbor the same eosinophilic inclusions that are present in other tissues in NIID, but the precise mechanism of the dynamic vascular changes that occur during encephalitic episodes is yet to be elucidated (14). Inclusions are also found in adipocytes, which raise the possibility of using skin biopsies to aid in diagnosing NIID in a less invasive fashion than a brain biopsy. This less invasive method was an important consideration for the family and patient in case 2 and was eventually implemented. It should be noted that similar intranuclear inclusions are also seen in adipocytes and other tissues in Fragile-X tremor ataxia syndrome (FXTAS), and in some situations, testing for a CGG repeat expansion in the FMR1 gene may be appropriate to rule out this condition which can cause a histological mimic (15). While the causative CGG repeat expansion in NOTCH2NLC and its association with NIID have been discovered, testing is not currently covered in most commercially available gene panels. It is likely that testing will be available in a more widespread fashion soon and will offer another method of diagnosing NIID without brain biopsy. It should be noted that NOTCH2NLC expansions are not universally associated with NIID as many non-Asian cases of NIID have been described that are NOTCH2NLC normal (16), and thus, a NOTCH2NLC mutation expansion may not be necessary to cause NIID, highlighting the importance of histopathology and corroborating clinical and radiological evidence.

Neuronal intranuclear inclusion disease remains an elusive diagnosis and should be considered in cases with atypical and recurrent episodes of acute encephalopathy, particularly when occurring on a background of progressive dementia. Furthermore, NIID should still be considered even in the absence of the more typical and well-described MRI finding of symmetric corticomedullary DWI hyperintensity, which may be more associated with the chronic phase of the disease. Given that less invasive methods of biopsy are now available, clinicians should have a low threshold to utilize skin biopsy in diagnosing this disease in the proper clinical context. Prognostic markers in this rare disease are generally lacking, as is guidance on the management during acute episodes of encephalopathy. However, increased awareness of this condition and its myriad clinical and imaging findings will promote a better understanding of the prevalence and phenotypic spectrum of this disease, which will aid in developing evidence-based guidance for its management.

## Data availability statement

The original contributions presented in the study are included in the article/Supplementary material, further inquiries can be directed to the corresponding author.

## Ethics statement

Written informed consent was obtained from the individual(s) for the publication of any potentially identifiable images or data included in this article.

## Author contributions

JB and DT wrote the first draft of the manuscript. JB, DT, JN, and DC wrote sections of the manuscript. AR, VG, and NF designed the figures. All authors contributed to the manuscript revision, read, and approved the submitted version.
