# Supplementary material for: Case report: Neuronal intranuclear inclusion disease presenting with acute encephalopathy
Source: Front Neurol. 2023 Jun 2;14:1184612. doi: 10.3389/fneur.2023.1184612 (PMC10272712; doi:10.3389/fneur.2023.1184612)
Supplement: Supplementary file 1 [file Table_1.DOCX]

Supplemental Material

Case 1:

Dosing for empiric antibiotics: IV acyclovir 450 mg q8 hours, ampicillin 2g q4 hours, Ceftriaxone 2gq day. Dosing for anti-seizure medication: Levetiracetam 2g BID and lacosamide 200 mg BID.

Dosing for concern adult onset MELAS: Arginine IV 500mg/kg per day for 5 days as well as Alpha Lipoic Acid 600 mg daily, Taurine 1g daily, Levocarnitine 330mg TID, Vitamin C 500 mg BID, and Coenzyme Q10 400mg daily

Case 2:

Genetic testing: NOTCH3, MNGIE, mitochondrial, Invitae leukodystrophy and genetic leukoencephalopathy panel were negative
